# Supplementary material for: Identification of Allergens in White- and Red-Fleshed Pitaya (Selenicereus undatus and Selenicereus costaricensis) Seeds Using Bottom-Up Proteomics Coupled with Immunoinformatics
Source: Nutrients. 2022 May 7;14(9):1962. doi: 10.3390/nu14091962 (PMC9134757; doi:10.3390/nu14091962)
Supplement: Supplementary file 1 [file nutrients-14-01962-s001.zip › nutrients-1687933-supplementary.pdf]

Supplementary Materials

Identification of Allergens in White- and Red-Fleshed Pitaya (*Selenicereus undatus* and *Selenicereus costaricensis*) Seeds Using Bottom-Up Proteomics Coupled with Immunoinformatics

Mengzhen Hao, XIJIRI, Ziyi Zhao and Huilian Che *

College of Food Science and Nutritional Engineering, China Agriculture University, Beijing 100083, China; mengzhen.hao@cau.edu.cn (M.H.); xijir0304@163.com (XIJIRI); ziyiiz@163.com (Z.Z.)

* Correspondence: chehuilian@cau.edu.cn

Table of contents

- **Table S1** Information of pitaya allergic patients.
- **Table S2** Information of primers for RT-PCR.
- **Table S3** Full list of the total protein from white-fleshed pitaya seeds protein band hits retrieved with MASCOT discoverer software screening.
- **Table S4** Full list of the total protein from red-fleshed pitaya seeds protein band hits retrieved with MASCOT discoverer software screening.
- **Table S5** Details for top 10% proteins identified via LC–MS/MS from white-fleshed pitaya seeds allergenicity predicted by three online platforms (Allermatchtm, Algpred 2.0, and AllerCatPro).
- **Table S6** Details for top 10% proteins identified via LC–MS/MS from red-fleshed pitaya seeds allergenicity predicted by three online platforms (Allermatchtm, Algpred 2.0, and AllerCatPro).
- **Table S7** CDSs for potential allergens in white- and red-fleshed pitaya seeds.
- **Supplement information S8: The distribution of identified peptides in the top 10% of protein identified via LC–MS/MS in excised gel spot from white-fleshed pitaya seeds.**
- **Supplement information S9: The distribution of identified peptides in the top 10% of protein identified via LC–MS/MS in excised gel spot from red-fleshed pitaya seeds.**

Table S1. Information of pitaya allergic patients.

Patient number	Age (years old)	Self-reporting symptoms	Symptoms occurring after eating white-fleshed or/and red-fleshed pitaya	Others
P1	23	Vomiting, diarrhea, and abdominal pain	White-fleshed pitaya	
P2	23	Vomiting and diarrhea	White-fleshed and red-fleshed pitaya	
P3	6	Wind mass rash, abdominal pain, and watery stools	Red-fleshed pitaya	Relieved after oral loratadine administration.
P4	33	Swelling of the right eyelid	Red-fleshed pitaya	
P5	14	Urticaria	White-fleshed and red-fleshed pitaya	

Table S2. Information of primers for RT-PCR.

Primers	Sequences (5'-3')
Cupin1 (Forward)	ATGATGGCACCCCATTTGAATC
Cupin1 (Reverse)	TCACTCAAACCCCATGACCATT
Hsp70 (Forward)	TTAATCAACCTCCTCAATCTTGGGA
Hsp70 (Reverse)	AAAGGTGAAGGTCCGGCG
Hsp sti 1 (Forward)	CATCTGACTTGGACAATGCC
Hsp sti 1 (Reverse)	ATGGCCGACGAAGCCAA

Table S3. Full list of the total protein from white-fleshed pitaya seeds protein band hits retrieved with MASCOT discoverer software screening.

N.	Description	Accession	Score	Mass (Da)	Peptides (Unique)	Coverage (%)
1	Uncharacterized protein (Fragment) OS=Beta vulgaris subsp. vulgaris OX=3555 GN=BVRB_027930 PE=3 SV=1	A0A0J8DSR1	158	26488	6(6)	24.1
2	Cupin type-1 domain-containing protein OS=Opuntia streptacantha OX=393608 PE=4 SV=1	A0A7C8ZNT3	143	18045	2(1)	12.7
3	Uncharacterized protein OS=Spinacia oleracea OX=3562 GN=SOVF_029540 PE=4 SV=1	A0A0K9RTZ6	137	65882	2(1)	4.7
4	Uncharacterized protein OS=Chenopodium quinoa OX=63459 PE=4 SV=1	A0A803LTG6	132	19434	4(4)	27.5
5	Heat shock protein 70 OS=Spinacia oleracea OX=3562 GN=HSP70-13 PE=2 SV=1	A0A1I9TK81	106	71808	3(3)	6.1
6	Proteasome subunit beta (Fragment) OS=Spinacia oleracea OX=3562 GN=SOVF_007620 PE=3 SV=1	A0A0K9S2G1	83	29745	1(1)	5.5
7	Histone H4 OS=Beta vulgaris subsp. vulgaris OX=3555 GN=BVRB_5g125910 PE=3 SV=1	A0A0J8BUP3	80	11402	1(1)	11.7
8	Formamidase OS=Opuntia streptacantha OX=393608 PE=4 SV=1	A0A7C9EWT0	77	52325	2(2)	6.1
9	VWFA domain-containing protein OS=Spinacia oleracea OX=3562 GN=SOVF_154600 PE=3 SV=1	A0A0K9QPX4	72	42327	1(1)	4.3
10	Uncharacterized protein OS=Opuntia streptacantha OX=393608 PE=4 SV=1	A0A7C8YYM2	66	16165	1(1)	4.8
11	PLAT domain-containing protein OS=Opuntia streptacantha OX=393608 PE=4 SV=1	A0A7C9CMR0	65	19550	1(1)	5.4
12	Proteasome subunit beta OS=Nepenthes alata OX=4376 GN=NaPSB1-1 PE=2 SV=1	A0A286T1W3	64	29511	2(2)	8.1
13	Tr-type G domain-containing protein OS=Beta vulgaris subsp. vulgaris OX=3555 GN=BVRB_2g046240 PE=3 SV=1	A0A0J8BGW2	60	94791	1(1)	1.4
14	ATP synthase subunit alpha OS=Beta vulgaris subsp. vulgaris OX=3555 GN=atpA PE=3 SV=1	A0A023ZQ87	58	55584	1(1)	2.6
15	Proteasome subunit beta OS=Opuntia streptacantha OX=393608 PE=3 SV=1	A0A7C9D7H1	56	22828	2(2)	10.2
16	Uncharacterized protein (Fragment) OS=Opuntia streptacantha OX=393608 PE=4 SV=1	A0A7C8YRL3	56	15770	2(2)	17.1
17	Uncharacterized protein OS=Beta vulgaris subsp. vulgaris OX=3555 GN=BVRB_9g209070 PE=3 SV=1	A0A0J8BPT8	49	24249	1(1)	4.5
18	D-xylose 1-dehydrogenase (NADP(+)) (Fragment) OS=Opuntia streptacantha OX=393608 PE=4 SV=1	A0A7C9AF84	48	42687	1(1)	2.6
19	Elongation factor Tu (Fragment) OS=Beta vulgaris subsp. vulgaris OX=3555 GN=BVRB_018790 PE=3 SV=1	A0A0J7YLM1	46	44450	2(2)	6.4
20	Dihydrolipoyllysine-residue succinyltransferase OS=Beta vulgaris subsp. vulgaris OX=3555 GN=BVRB_009840 PE=3 SV=1	A0A0J8B2D3	45	51446	1(1)	1.7
21	Ferritin OS=Beta vulgaris subsp. vulgaris OX=3555 GN=BVRB_2g044700 PE=3 SV=1	A0A0J8BHE5	45	29868	1(1)	3.4
22	Peroxidase OS=Tamarix hispida OX=189793 GN=POD2 PE=2 SV=1	C0KKH7	44	36304	1(1)	1.8

Continued Table S3

N.	Description	Accession	Score	Mass (Da)	Peptides (Unique)	Coverage (%)
23	Elongation factor 1-alpha OS=Hylocereus polyrhizus OX=1195597 GN=EF1A PE=2 SV=1	A0A249Y703	43	50180	1(1)	2.6
24	Uncharacterized protein (Fragment) OS=Opuntia streptacantha OX=393608 PE=4 SV=1	A0A7C9CHR1	43	23990	1(1)	5.3
25	Formylglycinamide ribonucleotide amidotransferase OS=Spinacia oleracea OX=3562 GN=SOVF_185900 PE=3 SV=1	A0A0K9QF72	40	149942	1(1)	0.7
26	Uncharacterized protein (Fragment) OS=Opuntia streptacantha OX=393608 PE=4 SV=1	A0A7C9ERX8	40	13642	1(1)	9.8
27	Uncharacterized protein OS=Chenopodium quinoa OX=63459 PE=4 SV=1	A0A803KW96	39	65071	1(1)	2.1
28	Uncharacterized protein OS=Spinacia oleracea OX=3562 GN=SOVF_024240 PE=3 SV=1	A0A0K9RVF6	39	36236	1(1)	3.8
29	Histone H2A OS=Beta vulgaris subsp. vulgaris OX=3555 GN=BVRB_015930 PE=3 SV=1	A0A0J8B158	38	15362	1(1)	6.2
30	Uncharacterized protein OS=Chenopodium quinoa OX=63459 PE=4 SV=1	A0A803LCC3	38	99945	1(1)	0.9
31	Annexin (Fragment) OS=Opuntia streptacantha OX=393608 PE=3 SV=1	A0A7C8Z5P5	37	36019	1(1)	2.8
32	Lactoylglutathione lyase OS=Opuntia streptacantha OX=393608 PE=3 SV=1	A0A7C9ED66	37	42702	1(1)	1.5
33	Uncharacterized protein OS=Chenopodium quinoa OX=63459 PE=4 SV=1	A0A803KPQ9	37	114105	1(1)	0.8
34	Uncharacterized protein OS=Chenopodium quinoa OX=63459 PE=4 SV=1	A0A803MK43	36	18155	1(1)	6.4
35	Uncharacterized protein OS=Spinacia oleracea OX=3562 GN=SOVF_117760 PE=4 SV=1	A0A0K9R339	36	101275	1(1)	0.7
36	Uncharacterized protein OS=Spinacia oleracea OX=3562 GN=SOVF_082480 PE=3 SV=1	A0A0K9RCF0	35	20634	1(1)	5.1
37	Ribulose bisphosphate carboxylase large chain (Fragment) OS=Persicaria punctata OX=137688 GN=rbcL PE=3 SV=1	A6YSW9	35	49327	1(1)	1.6
38	PPM-type phosphatase domain-containing protein OS=Spinacia oleracea OX=3562 GN=SOVF_154270 PE=4 SV=1	A0A0K9QRN3	34	52513	1(1)	3
39	Tubulin beta chain OS=Spinacia oleracea OX=3562 GN=SOVF_174120 PE=3 SV=1	A0A0K9QIW0	33	50836	1(1)	2.7
40	Uncharacterized protein OS=Spinacia oleracea OX=3562 GN=SOVF_047510 PE=3 SV=1	A0A0K9RPW9	33	35719	1(1)	3.1
41	Uncharacterized protein (Fragment) OS=Opuntia streptacantha OX=393608 PE=4 SV=1	A0A7C8ZTY5	33	12066	1(1)	5.7
42	Uncharacterized protein OS=Chenopodium quinoa OX=63459 PE=4 SV=1	A0A803KP77	33	29582	1(1)	4.2
43	Uncharacterized protein OS=Chenopodium quinoa OX=63459 PE=4 SV=1	tr|A0A803KM81|A0A803KM81_CHEQI	33	132058	1(1)	0.6
44	Uncharacterized protein OS=Chenopodium quinoa OX=63459 PE=4 SV=1	tr|A0A803LHI7|A0A803LHI7_CHEQI	33	84614	1(1)	2.5

Continued Table S3

N.	Description	Accession	Score	Mass (Da)	Peptides (Unique)	Coverage (%)
45	Uncharacterized protein (Fragment) OS=Opuntia streptacantha OX=393608 PE=3 SV=1	tr|A0A7C8ZY03|A0A7C8ZY03_OPUST	33	15483	1(1)	6.3
46	Proteasome subunit beta OS=Opuntia streptacantha OX=393608 PE=3 SV=1	tr|A0A7C9DYH2|A0A7C9DYH2_OPUST	33	29808	1(1)	3.3
47	Uncharacterized protein (Fragment) OS=Opuntia streptacantha OX=393608 PE=4 SV=1	tr|A0A7C8ZN49|A0A7C8ZN49_OPUST	32	24996	1(1)	4.6
48	Uncharacterized protein (Fragment) OS=Opuntia streptacantha OX=393608 PE=4 SV=1	tr|A0A7C9EA90|A0A7C9EA90_OPUST	32	10670	1(1)	6
49	RING-type E3 ubiquitin transferase OS=Opuntia streptacantha OX=393608 PE=4 SV=1	tr|A0A7C8YUS2|A0A7C8YUS2_OPUST	31	34317	1(1)	2.4
50	Uncharacterized protein OS=Spinacia oleracea OX=3562 GN=SOVF_021510 PE=4 SV=1	tr|A0A0K9RW14|A0A0K9RW14_SPIOL	31	24723	1(1)	5.2
51	Importin N-terminal domain-containing protein OS=Spinacia oleracea OX=3562 GN=SOVF_181840 PE=4 SV=1	tr|A0A0K9QGJ6|A0A0K9QGJ6_SPIOL	28	105505	1(1)	0.8
52	DUF4216 domain-containing protein (Fragment) OS=Beta vulgaris subsp. vulgaris OX=3555 GN=BVRB_016490 PE=4 SV=1	tr|A0A0J8B0Y1|A0A0J8B0Y1_BETVV	23	57613	1(1)	1
53	Uncharacterized protein (Fragment) OS=Opuntia streptacantha OX=393608 PE=4 SV=1	tr|A0A7C9D384|A0A7C9D384_OPUST	19	28239	1(1)	2.5

Table S4. Full list of the total protein from red-fleshed pitaya seeds protein band hits retrieved with MASCOT discoverer software screening.

N.	Description	Accession	Score	Mass (Da)	Peptides (Unique)	Coverage (%)
1	Proteasome subunit beta (Fragment) OS=Spinacia oleracea OX=3562 GN=SOVF_007620 PE=3 SV=1	A0A0K9S2G1	315	29745	4(1)	20.4
2	Uncharacterized protein (Fragment) OS=Opuntia streptacantha OX=393608 PE=4 SV=1	A0A7C8YRP5	283	15393	4(1)	37.8
3	Cupin type-1 domain-containing protein OS=Opuntia streptacantha OX=393608 PE=4 SV=1	A0A7C8ZNT3	274	18045	2(1)	12.7
4	Uncharacterized protein OS=Opuntia streptacantha OX=393608 PE=4 SV=1	A0A7C9B091	248	64965	5(5)	11
5	Heat shock protein 70 OS=Spinacia oleracea OX=3562 GN=HSP70-13 PE=2 SV=1	A0A1I9TK81	230	71808	6(6)	11
6	Actin 11 OS=Sesuvium portulacastrum OX=221166 PE=2 SV=1	A0A1L5JKA9	206	41929	6(4)	19.2
7	Ribosomal_L18e/L15P domain-containing protein OS=Beta vulgaris subsp. vulgaris OX=3555 GN=BVRB_4g076000 PE=3 SV=1	A0A0J8FEI6	182	20923	4(4)	26.2
8	Formamidase OS=Opuntia streptacantha OX=393608 PE=4 SV=1	A0A7C9EWT0	182	52325	3(3)	8.7
9	Uncharacterized protein OS=Spinacia oleracea OX=3562 GN=SOVF_029540 PE=4 SV=1	A0A0K9RTZ6	179	65882	2(1)	4.7
10	Uncharacterized protein (Fragment) OS=Opuntia streptacantha OX=393608 PE=4 SV=1	A0A7C8YRL3	170	15770	4(3)	34.2
11	Proteasome subunit beta OS=Opuntia streptacantha OX=393608 PE=3 SV=1	A0A7C9D7H1	164	22828	4(4)	15.6
12	Cupin_5 domain-containing protein OS=Opuntia streptacantha OX=393608 PE=4 SV=1	A0A7C9CU14	156	22754	1(1)	6.5
13	Uncharacterized protein OS=Opuntia streptacantha OX=393608 PE=3 SV=1	A0A7C8ZZS4	146	22123	4(1)	28
14	GTP-binding nuclear protein OS=Spinacia oleracea OX=3562 GN=SOVF_098770 PE=3 SV=1	A0A0K9R796	131	25564	4(3)	20.8
15	Uncharacterized protein OS=Chenopodium quinoa OX=63459 PE=4 SV=1	A0A803LTG6	129	19434	3(0)	19.9
16	Thioredoxin-dependent peroxiredoxin OS=Tamarix hispida OX=189793 GN=Prx2 PE=2 SV=1	I0CC94	123	30019	2(1)	10.9
17	Uncharacterized protein OS=Opuntia streptacantha OX=393608 PE=3 SV=1	A0A7C8Z5R6	122	22142	4(1)	22.8
18	Proteasome subunit beta OS=Nepenthes alata OX=4376 GN=NaPSB1-1 PE=2 SV=1	A0A286T1W3	121	29511	3(3)	11.7
19	Uncharacterized protein OS=Chenopodium quinoa OX=63459 PE=4 SV=1	A0A803LZK8	118	22897	2(0)	15.4
20	Uncharacterized protein OS=Chenopodium quinoa OX=63459 PE=4 SV=1	A0A803N503	114	27087	2(2)	10.1
21	Uncharacterized protein OS=Opuntia streptacantha OX=393608 PE=3 SV=1	A0A7C9DX00	110	17182	3(3)	13
22	Thioredoxin-dependent peroxiredoxin OS=Salicornia europaea OX=206448 PE=2 SV=1	A0A1D8H339	107	30203	2(1)	12
23	Uncharacterized protein OS=Opuntia streptacantha OX=393608 PE=3 SV=1	A0A7C9AUE0	105	16087	4(1)	37

Continued Table S4

N.	Description	Accession	Score	Mass (Da)	Peptides (Unique)	Coverage (%)
24	Uncharacterized protein OS=Opuntia streptacantha OX=393608 PE=4 SV=1	A0A7C8YYM2	105	16165	1(1)	4.8
25	Ribosomal_L18e/L15P domain-containing protein (Fragment) OS=Opuntia streptacantha OX=393608 PE=3 SV=1	A0A7C9CUT8	105	13740	2(1)	18.9
26	Uncharacterized protein OS=Opuntia streptacantha OX=393608 PE=4 SV=1	A0A7C9D682	96	24843	3(3)	17.9
27	Malate dehydrogenase OS=Spinacia oleracea OX=3562 GN=SOVF_175150 PE=3 SV=1	A0A0K9QKH5	93	36274	1(1)	4.7
28	S4 RNA-binding domain-containing protein OS=Spinacia oleracea OX=3562 GN=SOVF_105660 PE=3 SV=1	A0A0K9R566	92	23031	4(4)	21.3
29	Glutaminyl-tRNA synthetase OS=Opuntia streptacantha OX=393608 PE=3 SV=1	A0A7C8YIH5	89	91277	1(1)	1.6
30	Allene-oxide cyclase OS=Opuntia streptacantha OX=393608 PE=3 SV=1	A0A7C9EZI9	88	30285	3(3)	9.7
31	Uncharacterized protein OS=Opuntia streptacantha OX=393608 PE=4 SV=1	A0A7C9DW78	87	46521	1(1)	3.1
32	Peptidyl-prolyl cis-trans isomerase OS=Opuntia streptacantha OX=393608 PE=3 SV=1	A0A7C9DT77	87	25462	2(2)	10.3
33	Uncharacterized protein OS=Chenopodium quinoa OX=63459 PE=4 SV=1	A0A803MK43	86	18155	2(2)	6.4
34	Uncharacterized protein (Fragment) OS=Opuntia streptacantha OX=393608 PE=4 SV=1	A0A7C9ERX8	83	13642	2(2)	9.8
35	ATP synthase subunit alpha OS=Nepenthes ventricosa x Nepenthes alata OX=1744888 GN=atp1 PE=3 SV=1	A0A385Y4B7	77	55587	2(1)	4.3
36	SKP1-like protein OS=Spinacia oleracea OX=3562 GN=SOVF_115630 PE=3 SV=1	A0A0K9R207	77	18890	1(1)	7.2
37	UBX domain-containing protein OS=Opuntia streptacantha OX=393608 PE=4 SV=1	A0A7C9DHV8	76	50446	1(1)	3.1
38	Glyceraldehyde-3-phosphate dehydrogenase OS=Spinacia oleracea OX=3562 GN=SOVF_178150 PE=3 SV=1	A0A0K9QJL6	75	45322	1(1)	3.3
39	CN hydrolase domain-containing protein OS=Spinacia oleracea OX=3562 GN=SOVF_119350 PE=3 SV=1	A0A0K9R0Y0	74	37735	1(1)	4.3
40	Ribosomal_L16 domain-containing protein OS=Spinacia oleracea OX=3562 GN=SOVF_064690 PE=3 SV=1	A0A0K9R893	73	25709	1(1)	5.4
41	Ribosomal_L18e/L15P domain-containing protein OS=Beta vulgaris subsp. vulgaris OX=3555 GN=BVRB_6g141790 PE=3 SV=1	A0A0J8C427	72	29125	1(1)	4.1
42	MPN domain-containing protein (Fragment) OS=Opuntia streptacantha OX=393608 PE=3 SV=1	A0A7C9DSA4	72	34950	2(2)	7.4
43	Uncharacterized protein OS=Opuntia streptacantha OX=393608 PE=4 SV=1	A0A7C8Z9Z6	71	19831	1(1)	7.8
44	Uncharacterized protein OS=Spinacia oleracea OX=3562 GN=SOVF_074710 PE=4 SV=1	A0A0K9REU3	70	38307	2(2)	6.3
45	Uncharacterized protein OS=Spinacia oleracea OX=3562 GN=SOVF_028400 PE=3 SV=1	A0A0K9RU85	69	23734	1(1)	6.3
46	Superoxide dismutase OS=Opuntia streptacantha OX=393608 PE=3 SV=1	A0A7C8ZWU0	67	26282	3(3)	11.4

Continued Table S4

N.	Description	Accession	Score	Mass (Da)	Peptides (Unique)	Coverage (%)
47	Reticulon-like protein OS=Opuntia streptacantha OX=393608 PE=4 SV=1	A0A7C8ZF07	66	33220	2(2)	7.8
48	ATP synthase subunit alpha OS=Beta vulgaris subsp. vulgaris OX=3555 GN=atpA PE=3 SV=1	A0A023ZQ87	66	55584	1(1)	2.6
49	Uncharacterized protein OS=Chenopodium quinoa OX=63459 PE=4 SV=1	A0A803MYN6	65	17869	1(1)	8.9
50	Poly [ADP-ribose] polymerase (Fragment) OS=Spinacia oleracea OX=3562 GN=SOVF_124230 PE=4 SV=1	A0A0K9QZG2	65	92270	1(1)	1.6
51	Ubiquitin OS=Beta vulgaris subsp. vulgaris OX=3555 GN=BVRB_000430 PE=3 SV=1	A0A0J8B525	64	14906	1(1)	7
52	Acetohydroxy-acid reductoisomerase OS=Opuntia streptacantha OX=393608 PE=3 SV=1	A0A7C9ANA7	64	66498	1(1)	3
53	Uncharacterized protein OS=Spinacia oleracea OX=3562 GN=SOVF_102400 PE=3 SV=1	A0A0K9R865	64	60455	1(1)	2.6
54	Uncharacterized protein OS=Beta vulgaris subsp. vulgaris OX=3555 GN=BVRB_9g209070 PE=3 SV=1	A0A0J8BPT8	63	24249	2(2)	4.5
55	CSD domain-containing protein (Fragment) OS=Opuntia streptacantha OX=393608 PE=4 SV=1	A0A7C8ZG77	63	10757	1(1)	15.8
56	Uncharacterized protein OS=Beta vulgaris subsp. vulgaris OX=3555 GN=BVRB_005380 PE=3 SV=1	A0A0J8B417	62	44407	1(1)	3.2
57	Annexin OS=Spinacia oleracea OX=3562 GN=SOVF_164450 PE=3 SV=1	A0A0K9QNT1	62	36187	1(1)	3.5
58	Uncharacterized protein OS=Beta vulgaris subsp. vulgaris OX=3555 GN=BVRB_6g145160 PE=4 SV=1	A0A0J8C319	61	22671	1(1)	5.5
59	Ribosomal_S7 domain-containing protein OS=Beta vulgaris subsp. vulgaris OX=3555 GN=BVRB_3g050700 PE=3 SV=1	A0A0J8CS67	59	23395	2(2)	10.4
60	Adenosinetriphosphatase OS=Opuntia streptacantha OX=393608 PE=3 SV=1	A0A7C9CQ50	59	58332	2(2)	3.3
61	Elongation factor Tu OS=Opuntia streptacantha OX=393608 PE=3 SV=1	A0A7C9CTD2	58	53499	1(1)	2.3
62	14_3_3 domain-containing protein OS=Spinacia oleracea OX=3562 GN=SOVF_097310 PE=3 SV=1	A0A0K9R7S3	57	29569	1(1)	4.6
63	Uncharacterized protein OS=Chenopodium quinoa OX=63459 PE=4 SV=1	A0A803L2U0	56	29434	2(2)	8.8
64	Uncharacterized protein OS=Spinacia oleracea OX=3562 GN=SOVF_128330 PE=3 SV=1	A0A0K9QY28	55	42025	1(1)	2.6
65	40S ribosomal protein S4 OS=Beta vulgaris subsp. vulgaris OX=3555 GN=BVRB_004870 PE=3 SV=1	A0A0J8B7M5	54	29968	1(1)	4.2
66	Isocitrate lyase OS=Spinacia oleracea OX=3562 GN=SOVF_042350 PE=3 SV=1	A0A0K9RPI6	54	63996	3(3)	3.2
67	Malate synthase OS=Spinacia oleracea OX=3562 GN=SOVF_184210 PE=3 SV=1	A0A0K9QFS2	54	64615	1(1)	2.1
68	Uncharacterized protein OS=Chenopodium quinoa OX=63459 PE=4 SV=1	A0A803MNI3	54	18408	1(1)	4.8
69	Pyruvate kinase OS=Opuntia streptacantha OX=393608 PE=4 SV=1	A0A7C8Z1C6	54	21590	1(1)	7.6

Continued Table S4

N.	Description	Accession	Score	Mass (Da)	Peptides (Unique)	Coverage (%)
70	Uncharacterized protein (Fragment) OS=Opuntia streptacantha OX=393608 PE=4 SV=1	A0A7C8YQ02	53	18764	1(1)	8
71	Ribosomal_S17_N domain-containing protein OS=Spinacia oleracea OX=3562 GN=SOVF_049780 PE=3 SV=1	A0A0K9RPC5	53	17977	1(1)	6.9
72	Aryl-alcohol dehydrogenase (NADP(+)) OS=Opuntia streptacantha OX=393608 PE=3 SV=1	A0A7C8ZSD4	51	36894	2(2)	6.4
73	KH type-2 domain-containing protein OS=Beta vulgaris subsp. vulgaris OX=3555 GN=BVRB_010820 PE=3 SV=1	A0A0J8B2L2	51	27002	1(1)	5.4
74	Elongation factor Tu (Fragment) OS=Beta vulgaris subsp. vulgaris OX=3555 GN=BVRB_018790 PE=3 SV=1	A0A0J7YLM1	51	44450	1(1)	4.7
75	SBP-type domain-containing protein OS=Beta vulgaris subsp. vulgaris OX=3555 GN=BVRB_3g059840 PE=4 SV=1	A0A0J8CTR7	50	111979	1(1)	0.6
76	D-3-phosphoglycerate dehydrogenase OS=Opuntia streptacantha OX=393608 PE=3 SV=1	A0A7C9ER97	50	65170	1(1)	2.6
77	GOLD domain-containing protein OS=Spinacia oleracea OX=3562 GN=SOVF_044590 PE=3 SV=1	A0A0K9RP25	50	24852	1(1)	5.1
78	Elongation factor 1-alpha OS=Hylocereus polyrhizus OX=1195597 GN=EF1A PE=2 SV=1	A0A249Y703	49	50180	2(1)	5
79	Uncharacterized protein OS=Opuntia streptacantha OX=393608 PE=4 SV=1	A0A7C9CR18	49	48442	2(2)	4.7
80	D-xylose 1-dehydrogenase (NADP(+)) (Fragment) OS=Opuntia streptacantha OX=393608 PE=4 SV=1	A0A7C9AF84	48	42687	1(1)	2.6
81	Glyceraldehyde-3-phosphate dehydrogenase OS=Hylocereus polyrhizus OX=1195597 GN=G3P PE=2 SV=1	A0A249Y796	48	36839	2(2)	6.8
82	Uncharacterized protein OS=Spinacia oleracea OX=3562 GN=SOVF_021510 PE=4 SV=1	A0A0K9RW14	47	24723	1(1)	5.2
83	Ribos_L4_asso_C domain-containing protein OS=Spinacia oleracea OX=3562 GN=SOVF_207050 PE=3 SV=1	A0A0K9Q8R4	47	35669	1(1)	4
84	Uncharacterized protein OS=Opuntia streptacantha OX=393608 PE=3 SV=1	A0A7C9DCD2	47	13894	1(1)	6.7
85	PsbP domain-containing protein OS=Opuntia streptacantha OX=393608 PE=4 SV=1	A0A7C9D4B5	47	28412	1(1)	6.8
86	Uncharacterized protein OS=Spinacia oleracea OX=3562 GN=SOVF_123500 PE=3 SV=1	A0A0K9QZL7	46	55585	2(2)	4.3
87	Peroxidase OS=Tamarix hispida OX=189793 GN=POD2 PE=2 SV=1	C0KKH7	46	36304	1(1)	1.8
88	Ubiquitin thioesterase OTU1 OS=Spinacia oleracea OX=3562 GN=SOVF_156820 PE=4 SV=1	A0A0K9QPB3	46	23731	1(1)	5.3
89	TPR_REGION domain-containing protein (Fragment) OS=Opuntia streptacantha OX=393608 PE=4 SV=1	A0A7C8ZUV3	45	18155	1(1)	7.3
90	Ribosomal protein S19 OS=Drosera erythrorhiza OX=2005751 GN=rps19 PE=3 SV=1	A0A1Z1GBD9	45	10866	1(1)	8.6
91	Ubiquinone biosynthesis protein OS=Opuntia streptacantha OX=393608 PE=3 SV=1	A0A7C8YDY4	45	20731	1(1)	4.9
92	Cysteine proteinase inhibitor OS=Spinacia oleracea OX=3562 GN=SOVF_083420 PE=3 SV=1	A0A0K9RC70	45	32151	1(1)	3.2

Continued Table S4

N.	Description	Accession	Score	Mass (Da)	Peptides (Unique)	Coverage (%)
93	Uncharacterized protein OS=Chenopodium quinoa OX=63459 PE=4 SV=1	A0A803N5S8	45	118708	1(1)	1.3
94	Ferritin OS=Beta vulgaris subsp. vulgaris OX=3555 GN=BVRB_2g044700 PE=3 SV=1	A0A0J8BHE5	45	29868	1(1)	3.4
95	Germin-like protein OS=Beta vulgaris subsp. vulgaris OX=3555 GN=BVRB_001170 PE=3 SV=1	A0A0J8B8Q5	44	22570	1(1)	7.1
96	CCT-beta OS=Spinacia oleracea OX=3562 GN=SOVF_080100 PE=3 SV=1	A0A0K9RD41	44	57622	1(1)	2.3
97	Uncharacterized protein OS=Beta vulgaris subsp. vulgaris OX=3555 GN=BVRB_9g209130 PE=3 SV=1	A0A0J8BKL6	44	20978	1(1)	4.4
98	Malate dehydrogenase OS=Opuntia streptacantha OX=393608 PE=3 SV=1	A0A7C8ZW35	44	36113	1(1)	6.6
99	CCT-theta OS=Opuntia streptacantha OX=393608 PE=3 SV=1	A0A7C8ZZY3	44	59064	1(1)	2
100	GrpE protein homolog OS=Opuntia streptacantha OX=393608 PE=3 SV=1	A0A7C9E1C7	44	29986	1(1)	4.1
101	Peroxiredoxin OS=Beta vulgaris subsp. vulgaris OX=3555 GN=BVRB_010570 PE=3 SV=1	tA0A0J8B2B8	43	24516	1(1)	5
102	PRA1 family protein OS=Opuntia streptacantha OX=393608 PE=3 SV=1	A0A7C8YMH8	43	23315	1(1)	6.9
103	Non-specific serine/threonine protein kinase OS=Spinacia oleracea OX=3562 GN=SOVF_122500 PE=4 SV=1	A0A0K9R1K3	43	59199	1(1)	2.1
104	Biotin carboxylase OS=Spinacia oleracea OX=3562 GN=SOVF_065980 PE=4 SV=1	A0A0K9RHG8	43	58646	1(1)	2.8
105	Protein kinase domain-containing protein (Fragment) OS=Spinacia oleracea OX=3562 GN=SOVF_192390 PE=4 SV=1	A0A0K9QD95	43	46460	1(1)	1.9
106	Protein translocase subunit SecA OS=Spinacia oleracea OX=3562 GN=SOVF_169110 PE=3 SV=1	A0A0K9QMF2	42	114333	1(1)	1.3
107	Uncharacterized protein OS=Chenopodium quinoa OX=63459 PE=4 SV=1	A0A803L847	42	176812	1(1)	0.7
108	D-3-phosphoglycerate dehydrogenase OS=Spinacia oleracea OX=3562 GN=SOVF_126730 PE=3 SV=1	A0A0K9QYI9	41	57587	1(1)	3.1
109	Derlin OS=Spinacia oleracea OX=3562 GN=SOVF_187530 PE=3 SV=1	A0A0K9QGG3	41	27883	1(1)	4.9
110	Uncharacterized protein OS=Opuntia streptacantha OX=393608 PE=3 SV=1	A0A7C8YLZ9	41	13852	1(1)	10
111	Dihydrolipoyllysine-residue succinyltransferase OS=Beta vulgaris subsp. vulgaris OX=3555 GN=BVRB_009840 PE=3 SV=1	A0A0J8B2D3	41	51446	1(1)	1.7
112	Uncharacterized protein OS=Opuntia streptacantha OX=393608 PE=4 SV=1	A0A7C9DW29	40	36184	1(1)	4.2
113	Allene-oxide cyclase (Fragment) OS=Opuntia streptacantha OX=393608 PE=3 SV=1	A0A7C9APK0	40	22803	1(1)	6.7
114	Formylglycinamide ribonucleotide amidotransferase OS=Spinacia oleracea OX=3562 GN=SOVF_185900 PE=3 SV=1	A0A0K9QF72	40	149942	1(1)	0.7
115	Uncharacterized protein OS=Beta vulgaris subsp. vulgaris OX=3555 GN=BVRB_007550 PE=3 SV=1	A0A0J8D0R0	40	15837	1(1)	8.5

Continued Table S4

N.	Description	Accession	Score	Mass (Da)	Peptides (Unique)	Coverage (%)
116	Lactoylglutathione lyase OS=Opuntia streptacantha OX=393608 PE=3 SV=1	A0A7C9ED66	39	42702	1(1)	1.5
117	Uncharacterized protein OS=Opuntia streptacantha OX=393608 PE=4 SV=1	A0A7C9DMB2	39	22453	1(1)	7.3
118	Uncharacterized protein (Fragment) OS=Opuntia streptacantha OX=393608 PE=4 SV=1	A0A7C9CHR1	39	23990	2(2)	7.7
119	Ribosomal_L6e_N domain-containing protein OS=Opuntia streptacantha OX=393608 PE=3 SV=1	A0A7C8YCZ3	39	25970	1(1)	5.2
120	Uncharacterized protein OS=Opuntia streptacantha OX=393608 PE=3 SV=1	A0A7C9CUJ8	39	26378	1(1)	3.9
121	Lipocln_cytosolic_FA-bd_dom domain-containing protein OS=Spinacia oleracea OX=3562 GN=SOVF_207970 PE=3 SV=1	A0A0K9Q8I4	39	21646	1(1)	5.3
122	Aspartokinase OS=Spinacia oleracea OX=3562 GN=SOVF_023510 PE=3 SV=1	A0A0K9RXL4	38	100956	1(1)	0.9
123	Reticulon-like protein OS=Opuntia streptacantha OX=393608 PE=4 SV=1	A0A7C9DIY1	38	28770	1(1)	5.5
124	Uncharacterized protein OS=Spinacia oleracea OX=3562 GN=SOVF_192730 PE=3 SV=1	A0A0K9QD37	38	38000	1(1)	2
125	Malate dehydrogenase OS=Spinacia oleracea OX=3562 GN=SOVF_046560 PE=3 SV=1	A0A0K9RNG9	38	35899	1(1)	2.7
126	Uncharacterized protein OS=Opuntia streptacantha OX=393608 PE=4 SV=1	A0A7C8ZGD5	38	56683	1(1)	1.5
127	Uncharacterized protein OS=Spinacia oleracea OX=3562 GN=SOVF_107000 PE=4 SV=1	A0A0K9R4R0	36	181965	1(1)	0.4
128	Uncharacterized protein OS=Chenopodium quinoa OX=63459 PE=4 SV=1	A0A803KTW4	36	47501	1(1)	2.1
129	Uncharacterized protein (Fragment) OS=Opuntia streptacantha OX=393608 PE=4 SV=1	A0A7C9EID3	36	13180	1(1)	8
130	Uncharacterized protein OS=Chenopodium quinoa OX=63459 PE=4 SV=1	A0A803KPQ9	36	114105	1(1)	0.8
131	Uncharacterized protein (Fragment) OS=Opuntia streptacantha OX=393608 PE=3 SV=1	A0A7C8ZY03	36	15483	1(1)	6.3
132	PLAT domain-containing protein OS=Opuntia streptacantha OX=393608 PE=4 SV=1	A0A7C9CMR0	36	19550	1(1)	5.4
133	Uncharacterized protein (Fragment) OS=Opuntia streptacantha OX=393608 PE=3 SV=1	A0A7C8ZZR8	36	20251	1(1)	6.7
134	Uncharacterized protein OS=Opuntia streptacantha OX=393608 PE=4 SV=1	A0A7C8YTC3	36	47890	1(1)	2.7
135	Uncharacterized protein OS=Spinacia oleracea OX=3562 GN=SOVF_117760 PE=4 SV=1	A0A0K9R339	36	101275	1(1)	0.7
136	Uncharacterized protein OS=Beta vulgaris subsp. vulgaris OX=3555 GN=BVRB_3g065360 PE=3 SV=1	A0A0J8FG58	36	54945	1(1)	2.2
137	Cellulose synthase OS=Spinacia oleracea OX=3562 GN=SOVF_167560 PE=3 SV=1	A0A0K9QMU6	35	118591	1(1)	0.7
138	Usp domain-containing protein (Fragment) OS=Opuntia streptacantha OX=393608 PE=4 SV=1	A0A7C9DG99	35	14923	1(1)	6

Continued Table S4

N.	Description	Accession	Score	Mass (Da)	Peptides (Unique)	Coverage (%)
139	Alpha-1,4 glucan phosphorylase OS=Beta vulgaris subsp. vulgaris OX=3555 GN=BVRB_2g046340 PE=3 SV=1	A0A0J8E823	35	110302	1(1)	0.6
140	Adenosinetriphosphatase (Fragment) OS=Opuntia streptacantha OX=393608 PE=4 SV=1	A0A7C9DIR4	34	24101	1(1)	6.5
141	NAD(P)H dehydrogenase (quinone) OS=Spinacia oleracea OX=3562 GN=SOVF_195640 PE=4 SV=1	A0A0K9QC65	34	20094	1(1)	7.4
142	Uncharacterized protein (Fragment) OS=Spinacia oleracea OX=3562 GN=SOVF_207490 PE=3 SV=1	A0A0K9Q8M8	34	20562	1(1)	5.1
143	Malate synthase OS=Opuntia streptacantha OX=393608 PE=3 SV=1	A0A7C8ZIT7	34	46998	1(1)	2.4
144	Phosphoenolpyruvate carboxylase (Fragment) OS=Anredera baselloides OX=1038467 GN=ppc-1E1a PE=4 SV=1	A0A075IYP2	34	36088	1(1)	3.2
145	Protein-methionine-S-oxide reductase OS=Opuntia streptacantha OX=393608 PE=3 SV=1	A0A7C8YWM6	34	12347	2(2)	20.2
146	Uncharacterized protein OS=Chenopodium quinoa OX=63459 PE=4 SV=1	A0A803M627	34	22027	1(1)	5.8
147	Uncharacterized protein OS=Opuntia streptacantha OX=393608 PE=3 SV=1	A0A7C9DPF6	33	56110	1(1)	2.2
148	EngB-type G domain-containing protein OS=Spinacia oleracea OX=3562 GN=SOVF_207810 PE=3 SV=1	A0A0K9QA42	33	23645	1(1)	6.6
149	Uncharacterized protein (Fragment) OS=Opuntia streptacantha OX=393608 PE=4 SV=1	A0A7C8ZTY5	33	12066	1(1)	5.7
150	TCTP domain-containing protein (Fragment) OS=Opuntia streptacantha OX=393608 PE=3 SV=1	A0A7C9A5U1	33	12322	1(1)	8.3
151	4HBT domain-containing protein OS=Spinacia oleracea OX=3562 GN=SOVF_212720 PE=3 SV=1	A0A0K9Q7A9	33	17040	1(1)	3.8
152	Uncharacterized protein OS=Spinacia oleracea OX=3562 GN=SOVF_092000 PE=3 SV=1	A0A0K9RAQ0	33	17219	1(1)	8.8
153	Uncharacterized protein OS=Spinacia oleracea OX=3562 GN=SOVF_074910 PE=3 SV=1	A0A0K9RG77	33	286882	1(1)	0.7
154	PB1 domain-containing protein OS=Spinacia oleracea OX=3562 GN=SOVF_166830 PE=4 SV=1	A0A0K9QL33	33	85773	1(1)	0.8
155	Uncharacterized protein OS=Opuntia streptacantha OX=393608 PE=3 SV=1	A0A7C8YCZ0	32	27847	1(1)	4.9
156	Reverse transcriptase domain-containing protein OS=Beta vulgaris subsp. vulgaris OX=3555 PE=4 SV=1	F4NCJ4	32	157039	1(1)	1.1
157	60S ribosomal protein L13 OS=Spinacia oleracea OX=3562 GN=SOVF_142670 PE=3 SV=1	A0A0K9QV99	32	23811	1(1)	2.9
158	Uncharacterized protein (Fragment) OS=Opuntia streptacantha OX=393608 PE=4 SV=1	A0A7C8YK87	32	11461	1(1)	5.9
159	14_3_3 domain-containing protein OS=Opuntia streptacantha OX=393608 PE=3 SV=1	A0A7C9F4I6	32	23421	1(1)	7.8
160	Bifunctional dihydrofolate reductase-thymidylate synthase OS=Spinacia oleracea OX=3562 GN=SOVF_062630 PE=3 SV=1	A0A0K9RIF4	32	63193	1(1)	1.4
161	Uncharacterized protein OS=Spinacia oleracea OX=3562 GN=SOVF_131520 PE=4 SV=1	A0A0K9QX48	32	66773	1(1)	2.8

Continued Table S4

N.	Description	Accession	Score	Mass (Da)	Peptides (Unique)	Coverage (%)
162	Ribosomal_L6 domain-containing protein (Fragment) OS=Opuntia streptacantha OX=393608 PE=3 SV=1	A0A7C8ZLQ1	31	15674	1(1)	10.8
163	Phenylcoumaran benzylic ether reductase-like protein I (Fragment) OS=Cylindropuntia imbricata OX=701514 PE=2 SV=1	A0A3G4R8N0	31	32063	1(1)	4.1
164	Pyrophosphate--fructose 6-phosphate 1-phosphotransferase subunit alpha OS=Spinacia oleracea OX=3562 GN=PFP-ALPHA PE=3 SV=1	A0A0K9QFM3	30	68472	1(1)	1.3
165	Malectin_like domain-containing protein OS=Opuntia streptacantha OX=393608 PE=4 SV=1	A0A7C9DPM0	30	69601	1(1)	1.3
166	NTP_transferase domain-containing protein OS=Spinacia oleracea OX=3562 GN=SOVF_118350 PE=4 SV=1	A0A0K9R188	30	45858	1(1)	3.2
167	Tubulin alpha chain OS=Beta vulgaris subsp. vulgaris OX=3555 GN=BVRB_5g125100 PE=3 SV=1	A0A0J8E3G3	30	50783	1(1)	2.6
168	Uncharacterized protein OS=Chenopodium quinoa OX=63459 PE=4 SV=1	A0A803L2J9	30	181673	1(1)	0.4
169	Uncharacterized protein OS=Beta vulgaris subsp. vulgaris OX=3555 GN=BVRB_2g046040 PE=3 SV=1	A0A0J8E851	30	23784	1(1)	5.2
170	Heat shock protein 90 OS=Salicornia europaea OX=206448 PE=2 SV=1	L7QJJ0	29	79996	1(1)	0.9
171	Uncharacterized protein (Fragment) OS=Opuntia streptacantha OX=393608 PE=4 SV=1	A0A7C9A5J3|	28	13308	1(1)	13.9
172	Uncharacterized protein OS=Opuntia streptacantha OX=393608 PE=3 SV=1	A0A7C9CDJ2	28	13581	1(1)	6.8
173	40S ribosomal protein S7 OS=Spinacia oleracea OX=3562 GN=SOVF_134950 PE=3 SV=1	A0A0K9QW07	28	22112	1(1)	4.7
174	Purple acid phosphatase OS=Spinacia oleracea OX=3562 GN=SOVF_092830 PE=3 SV=1	A0A0K9R972	28	60725	1(1)	3
175	AAA domain-containing protein OS=Spinacia oleracea OX=3562 GN=SOVF_151020 PE=3 SV=1	A0A0K9QSR5	28	47213	1(1)	3.1
176	Uncharacterized protein OS=Spinacia oleracea OX=3562 GN=SOVF_002290 PE=3 SV=1	A0A0K9S456	26	17988	1(1)	5.4
177	Protein kinase domain-containing protein OS=Beta vulgaris subsp. vulgaris OX=3555 GN=BVRB_1g021560 PE=3 SV=1	A0A0J8BFG4	25	110090	1(1)	0.7
178	Proteasome subunit beta OS=Opuntia streptacantha OX=393608 PE=3 SV=1	A0A7C9DYH2	20	29808	1(1)	3.3

Table S5. Details for top 10% proteins identified via LC–MS/MS from white-fleshed pitaya seeds allergenicity predicted by three online platforms (Allermatchtm, Algpred 2.0, and AllerCatPro).

**Unpredicted top 10% protein**		**Prediction platform**			**Allergen information**	
**N.**	**Description**	**Allermatchtm**	**Algpred**	**AllerCatPro**	**Allergen code**	**Allergen source**
1	Uncharacterized protein (Fragment) OS=Beta vulgaris subsp. Vulgaris A0A0J8DSR1	-	-	no	-	-
2	Cupin type-1 domain-containing protein OS=Opuntia streptacantha A0A7C8ZNT3	+	+	week	Pollen allergen Coc n1	Cocos nucifera (Coconut)
3	Uncharacterized protein OS=Spinacia oleracea A0A0K9RTZ6	+	+	week	Pollen allergen Coc n1	Cocos nucifera (Coconut)
4	Uncharacterized protein OS=Chenopodium quinoa A0A803LTG6	-	-	no	-	-
5	Heat shock protein 70 OS=Spinacia oleracea A0A1I9TK81	+	+	strong	Tyr p 28	Tyrophagus putrescentiae (Storage mite)

Table S6. Details for top 10% proteins identified via LC–MS/MS from red-fleshed pitaya seeds allergenicity predicted by three online platforms (Allermatchtm, Algpred 2.0, and AllerCatPro)

**Unpredicted top 10% protein**		**prediction platform**			**Allergen information**	
**N.**	**Description**	**Allermatchtm**	**Algpred**	**AllerCatPro**	**Allergen code**	**Allergen source**
1	Proteasome subunit beta (Fragment) OS=Spinacia oleracea A0A0K9S2G1	-	-	**no**	-	-
2	Uncharacterized protein (Fragment) OS=Opuntia streptacantha A0A7C8YRP5	-	+	**no**	-	-
3	Cupin type-1 domain-containing protein OS=Opuntia streptacantha A0A7C8ZNT3	+	+	**week**	Pollen allergen Coc n1	*Cocos nucifera* (Coconut)
4	Uncharacterized protein OS=Opuntia streptacantha A0A7C9B091	+	-	**strong**	Major latex allergen Hev b 5 Gluten-like Q-repeats	*Hevea brasiliensis* (Para rubber tree (latex))
5	Heat shock protein 70 OS=Spinacia oleracea A0A1I9TK81	+	+	**strong**	Tyr p 28	*Tyrophagus putrescentiae* (Storage mite)
6	Actin 11 OS=Sesuvium portulacastrum A0A1L5JKA9	-	-	**no**	-	-
7	Ribosomal_L18e/L15P domain-containing protein OS=Beta vulgaris subsp. Vulgaris A0A0J8FEI6	-	-	**no**	-	-
8	Formamidase OS=Opuntia streptacantha A0A7C9EWT0	-	-	**no**	-	-
9	Uncharacterized protein OS=Spinacia oleracea A0A0K9RTZ6	+	+	**week**	Pollen allergen Coc n1 Gly m Bd 28K	*Cocos nucifera* (Coconut) *Glycine max* (Soybean)
10	Uncharacterized protein (Fragment) OS=Opuntia streptacantha A0A7C8YRL3	-	+	**no**	-	-
11	Proteasome subunit beta OS=Opuntia streptacantha A0A7C9D7H1	-	-	**no**	-	-

Continued Table S6

Unpredicted top 10% protein		prediction platform			Allergen information	
**N.**	**Description**	**Allermatchtm**	**Algpred**	**AllerCatPro**	**Allergen code**	**Allergen source**
12	Cupin_5 domain-containing protein OS=Opuntia streptacantha A0A7C9CU14	-	-	**no**	-	-
13	Uncharacterized protein OS=Opuntia streptacantha A0A7C8ZZS4	-	-	**no**	-	-
14	GTP-binding nuclear protein OS=Spinacia oleracea A0A0K9R796	-	-	**no**	-	-
15	Uncharacterized protein OS=Chenopodium quinoa A0A803LTG6	-	-	**no**	-	-
16	Thioredoxin-dependent peroxiredoxin OS=Tamarix hispida I0CC94	+	+	**week**	thiol peroxiredoxin	*Bombyx mori* (domestic silkworm)
17	Uncharacterized protein OS=Opuntia streptacantha A0A7C8Z5R6	-	-	**no**	-	-

Table S7. CDSs for potential allergens in white- and red-fleshed pitaya seeds.

CDS name	Sequences	Length (bp)	Location on the genome
Su-cupin1 Or *Sc-cupin1*	ATGATGGCACCCCATTTGAATCCAACGGCCACAGAGTATGCCG<br>TAGTTTTAAGCGGACGAGGAAACATCCAGGTAGTCTACCCAA<br>ACGGAAGCTCAGCGATGAGTGCCAGAGTCAGTGAAGGTGATG<br>TCTTCTGGATTCCAAAGTACTTCCCTTTCTGCCAGATTGCTTCC<br>AGGGCTGGCCCATTCGAGTTCTTGGGATTCACCACCTCAGCTC<br>ACCGGAACCGGCCCCAGTTCCTAGTGGGTGCCTCCTCAATCCT<br>GAACACCATGCGAGCCCCCGAACTTGCAACTGCTTTTGGCTTA<br>ACCGAAGAGAGGTTCGGCGAGATTGTGGGTGCTCAGAGAGA<br>GGCGGTCATTCTTCCGTCTCCTGCAGCTGCTCCACCCGATGGG<br>AAGGAGCGGTCAAAGTCGGAGGGGGAGGGGTCAAAATCCGA<br>GGTGGAGGAAAAGAACAAGCTAGAGGTGCCAAAGTTGACTA<br>GGACTTTAGGTCCAGATATGGTCATGGGTTTTGAGTGA	504	From 65759275 to 65759778

Su and *Sc* are the abbreviations for *Selenicereus undatus* (white-fleshed pitaya) and *Selenicereus costaricensis* (red-fleshed pitaya), respectively.

Continued Table S7

CDS name	Sequences	Length (bp)	Location on the genome
Su-hsp70<br>Or<br>*Sc-hsp70*	ATGGCCGGTAAAGGTGAAGGTCCGGCGATCGGAATCGATCTCGGAACGACATACTCATGCGTCGGTGTCTGG<br>CAGCATGATCGTGTTGAGATCATCGCCAATGATCAAGGAAATCGTACCACACCGTCTTATGTTGCTTTCACCGA<br>CACTGAGCGTTTGATCGGTGATGCTGCTAAGAATCAGGTCGCCATGAATCCTACCAACACCGTATTGATGCTA<br>AGAGGCTTATCGGAAGGAGGTTCAGTGACTCATCTGTTCAGAATGACATGAAACTCTGGCCATTTAAGGTCAT<br>TGCTGGTGCTGGTGACAAGCCTATGATTGTGGTAACATATAAGGGTGAGGAGAAGCAATTTGCAGCTGAAGA<br>GATCTCTTCAATGGTCCTCACAAAGATGAAAGAGATTGCGGAGGCCTACCTTGGCTCAACGGTGAAAAACGC<br>TGTTGTCACTGTTCCTGCTTACTTCAACGATTCCCAGCGTCAAGCTACAAAGGATGCTGGTGTCATTGCTGGC<br>TTGAATGTTATGCGAATCATCAACGAGCCAACCGCTGCTGCTATTGCCTATGGTCTTGACAAGAAGGCCACCA<br>GCATTGGCGAGAAGAACGTCCTGATCTTTGATCTTGGTGGTGGTACCTTTGATGTCTCGTTGCTTACTATTGAG<br>GAGGGTATCTTTGAGGTGAAAGCCACAGCTGGAGACACCCATCTTGGTGGTGAGGATTTTGACAACCGAATG<br>GTAAACCATTTTGTCCAGGAGTTCAAGAGAAAGCACAAGAAGGATATCAGCGGCAGCCCAAGGGCTCTTAGG<br>AGGCTGAGGACTGCATGTGAGAGGGCAAAGAGGACTCTCTCATCCACTGCCCAAACCACCATTGAAATTGAC<br>TCCCTCTATGAGGGTGTTGATTTCTACACCACCATCACCCGTGCTCGTTTTGAGGAGCTGAACATGGATTTGTT<br>TAGGAAATGTATGGAGCCAGTGGAGAAGTGTTTGAGGGATGCTAAGATGGACAAGAGTAGCGTGCATGATGT<br>TGTCCTTGTTGGAGGATCCACTAGGATTCCCAAGGTCCAGACGCTGTTGCAGGACTTCTTCAACGGCAAGGA<br>GCTGTGCAAAAGCATTAACCCAGATGAGGCGGTTGCCTATGGTGCCGCTGTGCAAGCTGCCATCTTGAGTGGT<br>GAAGGGAACGAGAAGGTCCAGGACCTCCTGTTGCTCGATGTCACTCCTCTCTCCCTTGGTTTGGAAACCGCT<br>GGTGGAGTCATGACTGTGTTGATCCCTAGGAATACCACCATTCCAACCAAGAAAGAGCAGGTCTTCTCAACG<br>TACTCGGACAACCAGCCGGGTGTGTTGATCCAGGTTTACGAGGGTGAGAGAACTCGGACACGGGATAACAAC<br>TTGCTGGGAAAGTTTGAGCTTTCCGGCATCCCTCCCGCCCCTAGAGGTGTACCCCAGATTACAGTGTGCTTTG<br>ACATTGATGCCAATGGTATCTTGAATGTTTCTGCTGAGGACAAGACTACTGGGCAGAAGAACAAGATCACCAT<br>CACCAACGACAAGGGCAGGTTGTCAAAAGAAGAGATCGAGAAGATGGTCCAGGAGGCTGAGAAGTACAAG<br>ACTGAAGATGAGGAGCACAAGAAGAAGGTGGAGGCAAAGAATGCTTTGGAGAACTACGCCTACAACATGAG<br>GAATACTGTAAGGGATGAGAAGATTGGTGCGAAGTTGGCAGAGGCCGACAAGAAGAAGATCGAGGATGCTAT<br>CGACAATGCCATCCAGTGGTTGGATAATAATCAGCTTGCTGAGGCTGATGAATTTGAGGACAAAATGAAGGAG<br>CTCGAGAGTATCTGCAACCCCATCATTGCCAAGATGTACCAGGGTGCTGCTGGGGGTGACATGGGTGGTGCC<br>ATGGATGATGATGGCCCCTCTGCTGGTGCAAGTGGCGCTGGTCCCAAGATTGAGGAGGTTGATTAA	1949	From 32343233 to 32344969 and from 32345659 to 32345870

Su and *Sc* are the abbreviations for *Selenicereus undatus* (white-fleshed pitaya) and *Selenicereus costaricensis* (red-fleshed pitaya), respectively.

Continued Table S7

CDS name	Sequences	Length (bp)	Location on the genome
Sc- hsp sti 1	ATGGCTGAAGAGGCCAAAGCCAAAGGCAACGCCGCCTTCGC<br>CGCCGGCAACTACACGGAGGCCATTAAGCACTTCACGGAGGG<br>GATCATCCTGGCCCCCACCAACCACGTCCTCTACTCCAACAGA<br>TCCGCCGCATACGCCTCCCTCCACAACTACTCCGACGCCCTCT<br>CCGATGCCAAGAAGACGGTCGACCTCAAGCCCGACTGGTCCA<br>AGGGCTACAGCCGCCTTGGCGCCGCCTATCTCGGCCTCCATAA<br>CTACTCCGAAGCCATCGCCGCCTACAAGCATGGCCTTGAAATC<br>GACCCCAATAACGAGGCTCTGAAATCCGGCTTAGCCGATGCG<br>CAAGCAGCCGCCGCCCCCCGAGCCAGGTCGACCTCGCCGCCT<br>TCCCCCTTCGGCGACGTCTTCTCCGGGCAGGAGATGTGGGCC<br>AAGCTCACGGCGGACCCCACCACTCGGGCATTCCTCCAGCAG<br>CCTGATTTCGTCAGCATGATGCAGGACATTCAGAAGAACCCTA<br>ATAATCTGAATCTATACTTGAAGGATCAGAGGGTTATGCAGGC<br>TCTTGGGGCTCTTTTGAATGTCAAATTGAGGACTGCAACTGAG<br>GATATGGAAGTGGAACAACCTTTTTCTGAGGAGGAGCCGGTG<br>AAGAAGCCCGAGCCAGAGCCTCGACGGGAGCCGGAGCCTGA<br>GCCCATCGAGCTGGCGGAGGAGGAGAGAGAAAGGAAGGAA<br>AAGAAGGCATTGGCCCAGAAGGAGAAAGAGGCTGGCAATGC<br>TGCTTATAAGAAGAAGGATTTTGAGGCTGCTATTCAGCATTAC<br>ACCAAGGCCATTGAATTGGATGATGAGGATATTTCCTTCTTCA<br>CTAATCGTGCTGCTGTTTACTTGGAGATGGGAAAGGTACGATG<br>AGTGTATCAAAGACTGTGACAAAGCTGTTGAAAGAGGAAGG<br>GAACTTCGATCAGACTACAAGATGATAGCAAGGGCTTTGACC<br>AGGAAAGGAACTGCCTTGGTTAAGATGGCAAAGACTTCAAAG<br>GACTTTGAGACGGCTATTGAAGCTTTTCAGAAAGCTCTCACTG<br>AGCATCGCAATCCTGATACATTGAAAAAGTTGAACGATGCTGA<br>AAGAGCAAAGAAAGAACTAGAGCAGCAGGAGTACTTTGATC<br>CAAAAATAGCCGATCAGGAACGTGAAAAAGGAGGCATATAGC<br>AACAGAGCTGCATGTTACACGAAATTGGGGGCATTGCCAGAA<br>GCTCTAAAAGATGCAGAGAAGTGTATCAAGCTTGATCCTACAT<br>TTGTGAAGGGATATACTAGGAAGGGTGCTGCCCAGTTTTTGAT<br>GAAGGAGCATGATAAAGCATTGGAAACCTATCAAGAAGGGCT<br>TAAGCATGATCCCAACAACCAGGAATTGTTGGATGGGGTGCG<br>GAGGTGTGTCAGGTATTGATTGACTTCCAAGAGAATCCAAAG<br>GCAGCACAAGAGCATATGAAGAACCCAATGGTGATGAACAAG<br>ATCCAGAAGCTGGTCAGCGCTGGAATTGTCCAAGTCAGATGA	1519	From 8614836 to 8615721, from 8616533 to 8616816, from 8617196 to 8617427, from 8617842 to 8617958

Su and *Sc* are the abbreviations for *Selenicereus undatus* (white-fleshed pitaya) and *Selenicereus costaricensis* (red-fleshed pitaya), respectively.

Supplement information S8. The distribution of identified peptides in the top 10% of protein identified via LC–MS/MS in excised gel spot from white-fleshed pitaya seeds (The peptides coverages have been marked with highlight).

>tr|A0A0J8DSR1|A0A0J8DSR1_BETVV Uncharacterized protein (Fragment) OS=Beta vulgaris subsp. vulgaris OX=3555 GN=BVRB_027930 PE=3 SV=1
SIRVAPLRAMCDDEVQALVVDNGSGMVKAGFAGDDAPRAVFPSIVGRPRHTGIMVGATQ
KDAYVGDEAQAKRGILTLKYPIEHGIVTNWDDMEKIWHHTFYNELRVAPEEHPVLLTEAP
LNPKANREKMTQIMFETFNTPAMYVAIQAVLSLYASGRTTGIVLDCGDGVSHTVPIYEGYA
LPHAILRLDLAGRDLTDYLMKILTERGYSFTTTAEREIVRDIKEKLAYCALDFDTEM

>tr|A0A7C8ZNT3|A0A7C8ZNT3_OPUST Cupin type-1 domain-containing protein OS=Opuntia streptacantha OX=393608 PE=4 SV=1
MMAPHLNPMATEYGIVLSGRGKIQVVYPNGSSAMNARVSEGDVFWIPKYFPFCQIASRTG
PFEFFGFTTSAHRNRPQFLVGASSILNAMRGPELATAFGLTEERFDEIVSAQREAVILPSPAA
APPDESGGSKSEQEGLDSETEKKKTVVPKLIRTLGPAMVMGFE

>tr|A0A0K9RTZ6|A0A0K9RTZ6_SPIOL Uncharacterized protein OS=Spinacia oleracea OX=3562 GN=SOVF_029540 PE=4 SV=1
MGRLKGVLLVALVVCYAMQVVVSQSYEERRRERELMSPEEEERRAKEEDWRRREKTAEE
ERRHRRSWEEEEEEEEERGRRKWDEDEEEQEEEERGRQPRPKPRPEPYRPGGRGGEGMF
VLRDSKKVISTQAGEMRVVRGYGGKIVENPLDIGFITMEPRSLFVPQYLDSSLIIFLRRGEA
KLGFIYDDELSERQLKMGDVYRIPAGSTFYIVNTGETQRLHIICSIDPSEGLGFSTFQSFFIGG
GTNPVSVLAGFDPETLSTAFNVSIGELRGFMTGQDAGPIIFADTTHSPSLWANFLKVKGEER
LERLKTVAESGEQKEEEEEEPRKSWWNIFDSLIGSENKKGEKKGDTRTGSSPDSYNLLDRE
PSYRNNYGWSIAVDKHEYKPLKKSGIGVYLVNLTAGSMMAPHVNPMATEYGVVLSGVGT
IQVVYPNGTSAMNTNVKEGDVFWIPRYFPFCQIASRSGSFEFFGFTTSAHRNRPQFLVGAS
SILRSMRGPEFATAFGLTEDRYNEIVDAQREALILPSPSAASGDTREFEEKEQSSQEKKPESK
EQEKEKPGIEKVPKFMRTLGPEMIMGFE

>tr|A0A803LTG6|A0A803LTG6_CHEQI Uncharacterized protein OS=Chenopodium quinoa OX=63459 PE=3 SV=1
MRQCLIYDSPEADARLIGIEYIVSEDLFMTLPDAEKRLWHSHEYEVKSGVLFLPGVPGAM
QRPDLAKVAQTYGKTIHFWQVDRGDNLPLGLPQIMMALTRDGQLYTHLASDVEKKYNV
NFEKERENRAYMKGLEHGIHPLANAEGKGLRTELREVDCHGGAGAHSTPRVFV

>tr|A0A1I9TK81|A0A1I9TK81_SPIOL Heat shock protein 70 OS=Spinacia oleracea OX=3562 GN=HSP70-13 PE=2 SV=1
MSGKGEGPAIGIDLGTTYSCVGVWQHDRVEIIANDQGNRTTPSYVAFTDTERLIGDAAKN
QVAMNPTNTVFDAKRLIGRRVSDPSVQADMKLWPFKVIPGPADKPMIVVNYKGEEKQFS
AEEISSMVLTKMKEIAEAFLGTTIKNAVVTVPAYFNDSQRQATKDAGVISGLNVMRIINEPT
AAAIAYGLDKKASSSGEKHVLIFDLGGGTFDVSLLTIEEGIFEVKATAGDTHLGGEDFDNR
MVNHFVQEFRRKHKKDISGNPRSLRRLRTACERAKRTLSSTAQTTIEIDSLYEGVDFYTTIT
RARFEELNMDLFRKCMEPVEKCLRDAKMDKGNVDDVVLVGGSTRIPKVQQLLQDFFNG

KELCKSINPDEAVAYGAAVQAAILSGEGNEKVQDLLLLDVTPLSLGLETAGGVMTVLIPRN
TTIPTKKEQVFSTYSDNQPGVLIQVFEGERTRTRDNNLLGKFELTGIPPAPRGVPQINVCFDI
DANGILNVSAEDKTTGQKNKITITNDKGRLSKEEIEKMVQEAERYKSEDEEHKKKVEAKN
ALENYAYNMRNTIKDDKINSKLDAADKKKIEEAIEQAIQWLDGNQLAEADEFDDKMKEL
EGICNPIIAKMYQGGAGAGPDMGGTMDEDAPSAGGASGAGPKIEEVD

Supplement information S9. The distribution of identified peptides in the top 10% of protein identified via LC–MS/MS in excised gel spot from red-fleshed pitaya seeds (The peptides coverages have been marked with highlight).

>tr|A0A0K9S2G1|A0A0K9S2G1_SPIOL Proteasome subunit beta (Fragment) OS=Spinacia oleracea OX=3562 GN=SOVF_007620 PE=3 SV=1
VLCDLHLYSPLSSFPFHHLQNRPISLQIPLSLSPQHTKMEDLGLNAPHSMGTTIIGVTYKDG
VILGADSRTSTGVYVANRASDKITQLTDNVYVCRSGSAADSQIVSDYVRYFLHQHTIQLGQ
PATVKVAANLVRLLAYGNKDTLQTGMIVGGWDKYEGGKIYGIPLGGTIIEQPFSIGGSGSS
YLYGFLDQAWKDGMSKDEAEELVKKAVSLAIARDGASGGVVRTVIINEEGVTRNFYPGD
QLPLWHEELEPQNSLLDIWGAAAASPVPMTE

>tr|A0A7C8YRP5|A0A7C8YRP5_OPUST Uncharacterized protein (Fragment) OS=Opuntia streptacantha OX=393608 PE=4 SV=1
VYVCRSGSAADSQIVSDYVRYFLHQHTIQLGQPATVKVAANLVRLLAYNNKNMLQTGLIV
GGWDKYEGGKIYGIPLGGTIIEQPFAIGGSGSSYLYGFFDQAWREGMTKEEAEQLVVKAVS
LAIARDGASGGVVRTVVINSEG

>tr|A0A7C8ZNT3|A0A7C8ZNT3_OPUST Cupin type-1 domain-containing protein OS=Opuntia streptacantha OX=393608 PE=4 SV=1
MMAPHLNPMATEYGIVLSGRGKIQVVYPNGSSAMNARVSEGDVFWIPKYFPFCQIASRTG
PFEFFGFTTSAHRNRPQFLVGASSILNAMRGPELATAFGLTEERFDEIVSAQREAVILPSPAA
APPDESGGSKSEQEGLDSETEKKKTVVPKLIRTLGPAMVMGFE

>tr|A0A7C9B091|A0A7C9B091_OPUST Uncharacterized protein OS=Opuntia streptacantha OX=393608 PE=4 SV=1
MADEAKAKGNAAFSAGNYTEAIKHFTEAINLAPTNHVLYSNRSAAYASLHNYSDALSDA
KKTVELKADWSKGYSRLGAAYLGLHQYSDAIAAYKQGLEIDPNNEALKSGLADAQAAA
RAKSTPLPSPFGDVFSGPEMWAKLTADPATRAFLQQPDFVSMMQDIQKNPSNLNLYLKDQ
RVMQALGVLLNVKLRTAPEDMEVPPAEELPLKKEEPAKKPEPKPQPQPEPEPEPVAEEEKE
KKERKALAQKEKEAGNAAYKKKDFETAIQHYTKAIELDDEDISFLTNRAAVYLEMGKYD
ECIKDCDKAVERGRELRSDFKMIARALTRKGTAFVKMAKTSKDYEPAIEAFQKALTEHRN
PDTLKKLNEAGRAKKELEQQEYFDPKIADEEREKGNEYFKEQKYPEAVKHYTEAIKRNPE
DPKAYSNRAACYTKLGALPEGLRDAEKCIELDPTFVKGYTRKGAVQFFMKEYDKALETY
QEGLKLDPNNQELLDGVRRCVEQINKASRGDLSPEELKERQAKAMQDPEIQTILTDPVMR
QVLIDFQENPKAAQEHMKNPMVMNKIQKLVSAGIVQVR

>tr|A0A1I9TK81|A0A1I9TK81_SPIOL Heat shock protein 70 OS=Spinacia oleracea OX=3562 GN=HSP70-13 PE=2 SV=1
MSGKGEGPAIGIDLGTTYSCVGVWQHDRVEIIANDQGNRTTPSYVAFTDTERLIGDAAKN
QVAMNPTNTVFDAKRLIGRRVSDPSVQADMKLWPFKVIPGPADKPMIVVNYKGEEKQFS
AEEISSMVLTKMKEIAEAFLGTTIKNAVVTVPAYFNDSQRQATKDAGVISGLNVMRIINEPT
AAAIAYGLDKKASSSGEKHVLIFDLGGGTFDVSLLTIEEGIFEVKATAGDTHLGGEDFDNR
MVNHFVQEFRRKHKKDISGNPRSLRRLRTACERAKRTLSSTAQTTIEIDSLYEGVDFYTTIT

RARFEELNMDLFRKCMEPVEKCLRDAKMDKGNVDDVVLVGGSTRIPKVQQLLQDFFNG
KELCKSINPDEAVAYGAAVQAAILSGEGNEKVQDLLLLDVTPLSLGLETAGGVMTVLIPRN
TTIPTKKEQVFSTYSDNQPGVLIQVFEGERTRTRDNNLLGKFELTGIPPAPRGVPQINVCFDI
DANGILNVSAEDKTTGQKNKITITNDKGRLSKEEIEKMVQEAERYKSEDEEHKKKVEAKN
ALENYAYNMRNTIKDDKINSKLDAADKKKIEEAIEQAIQWLDGNQLAEADEFDDKMKEL
EGICNPIIAKMYQGGAGAGPDMGGTMDEDAPSAGGASGAGPKIEEVD

>tr|A0A1L5JKA9|A0A1L5JKA9_SESPO Actin 11 OS=Sesuvium portulacastrum OX=221166 PE=2 SV=1
MCDDVQALVIDNGSGMCKAGFAGDDAPRAVFPSIVGRPRHTGVMVGMGQKDSYVGDEA
QSKRGILTLKYPIEHGIVTNWDDMEKIWHHTFYNELRVAPEEHPVLLTEAPLNPKANREK
MTQIMFETFNTPAMYVAIQAVLSLYASGRTTGIVMDSGDGVTHTVPIYEGYALPHAILRLD
LAGRDLTDYLMKILTERGYSFTTTAEREIVRDIKEKLAYVALDFEQEMQTAASSSSLEKSYE
LPDGQVITIGNERFRCPEALFQPSFLGMEAAGVHETTYNSIMKCDVDIRKDLYGNVVLSG
GSTMFPGIADRMQKELTALAPSTIKIKIIAPPERKYSVWIGGSILASLSTFQQMWISKEEYDE
SGPSIVHRKCF

>tr|A0A0J8FEI6|A0A0J8FEI6_BETVV Ribosomal_L18e/L15P domain-containing protein OS=Beta vulgaris subsp. vulgaris OX=3555 GN=BVRB_4g076000 PE=3 SV=1
MGIDLKAGGKSKKTKRTAPKSNDVYLKLLVKLYRFLVRRTGSSFNGVVLKRLFMSKINKA
PLSLSRLITYMKGKEDKIAVIVGAVTDDIRVYEVPALKVTALRFTETARARIEKAGGECLTF
DQLALRAPLGQNTVLLRGPKNAREAVKHFGKAPGVPHSHTKPYVRANGRKFERARGRR
NSRGYRV

>tr|A0A7C9EWT0|A0A7C9EWT0_OPUST Formamidase OS=Opuntia streptacantha OX=393608 PE=4 SV=1
NTLARSERPSIPRSPEGTKEMATSYGARTVIAVDVTKKPWEQKPPLHNRWHPDIPPVVAVK
AGEVFRVEMVDFSGGGITKDYTAEDVKYADPSVVHYLSGPIKVCDEAGVPAKPGDLLAVE
ICNLGPLPGDEWGYTATFDRENGGGFLTDHFPCATKAIWYFEGIYAYSPHIPGVRFPGLTHP
GIIGTAPSKQLLDIWNERERDVEENGLQSLKLCEVLHSRPLANLPSPKGCLLGKIEKGTPE
WEKIAREAARTIPGRENGGNCDIKNLSRGSKIYLPVFVEGANLSTGDMHFSQGDGEVSFC
GAIEMSGFLELKCDIIRGGMKEYLTPMGPTPLHVNPIFEIGPVEPRFSEWLVFEGISVDESGR
QHFLDASIAYKRAVLNAIDYLSKFGYSKEQVYLLLSCCPCEGRISGIVDAPNAVATLAIPTSI
FDQDIRPKTSKVPVGPRLVRKADVLKCTYDGHLPVTRNPAASS

>tr|A0A0K9RTZ6|A0A0K9RTZ6_SPIOL Uncharacterized protein OS=Spinacia oleracea OX=3562 GN=SOVF_029540 PE=4 SV=1
MGRLKGVLLVALVVCYAMQVVVSQSYEERRRERELMSPEEEERRAKEEDWRRREKTAEE
ERRHRRSWEEEEEEEEERGRRKWDEDEEEQEEEERGRQPRPKPRPEPYRPGGRGGEGMF
VLRDSKKVISTQAGEMRVVRGYGGKIVENPLDIGFITMEPRSLFVPQYLDSSLIIFLRRGEA
KLGFIYDDELSERQLKMGDVYRIPAGSTFYIVNTGETQRLHIICSIDPSEGLGFSTFQSFFIGG
GTNPVSVLAGFDPETLSTAFNVSIGELRGFMTGQDAGPIIFADTTHSPSLWANFLKVKGEER
LERLKTVAESGEQKEEEEEEPRKSWWNIFDSLIGSENKKGEKKGDTRTGSSPDSYNLLDRE
PSYRNNYGWSIAVDKHEYKPLKKSGIGVYLVNLTAGSMMAPHVNPMATEYGVVLSGVGT

IQVVYPNGTSAMNTNVKEGDVFWIPRYFPFCQIASRSGSFEFFGFTTSAHRNRPQFLVGAS
SILRSMRGPEFATAFGLTEDRYNEIVDAQREALILPSPSAASGDTREFEEKEQSSQEKKPESK
EQEKEKPGIEKVPKFMRTLGPEMIMGFE

>tr|A0A7C8YRL3|A0A7C8YRL3_OPUST Uncharacterized protein (Fragment) OS=Opuntia streptacantha OX=393608 PE=4 SV=1
CRSGSAADSQVVSDYVRYFLHQHTIQLGQPATVKVAANLVRLLAYNNKNMLQTGLIVGG
WDKYDGGKIYGIPLGGTLIEQPFAIGGSGSTYLYGFFDQAWKEGMTKDEAEQLVVKAVSL
AIARDGASGGVVRTVIINSEGVTRNFY

>tr|A0A7C9D7H1|A0A7C9D7H1_OPUST Proteasome subunit beta OS=Opuntia streptacantha OX=393608 PE=3 SV=1
MECVFGLVGNGFAIIAADTSAVHSILVHKSNEDKIMILDSHKLMGASGESGDRVQFTEYIQ
KNVALYQFRNGIPLTTAAAANFTRGELATALRKSPYFVNILLAGYDKETGPSLYFIDYIATL
HKVDKGAFGYGSYFALSMMDRHYHSGMSVEEAIDLVDKCIVEIRSRLVVAPPNFVIKIVDE
KGAREYGWRESVKDTTFPRVS

>tr|A0A7C9CU14|A0A7C9CU14_OPUST Cupin_5 domain-containing protein OS=Opuntia streptacantha OX=393608 PE=4 SV=1
EREREREREREMVGTSQIVEKLNLKAHVEGGYFHETFRDHSITLSKSHLPPQYKVDRPVST
AIYFLLPAGSVSKLHRIPMAETWHFYLGEPIMIVELNEKDASVKLTCLGPNLMENQQPQYT
VPPNVWFGSFPTKDISISPNGVATKAEPRDPETHFSLVGCTCAPAFQFEDFELAKRSELVSLF
PNYESLISLLTSSD

>tr|A0A7C8ZZS4|A0A7C8ZZS4_OPUST Uncharacterized protein OS=Opuntia streptacantha OX=393608 PE=3 SV=1
MFLVDWFYGILASLGLWQKEAKILFLGLDNAGKTTLLHMLKDERLVQHQPTQYPTSEELS
IGKIKFKAFDLGGHQIARRVWKDYYAKVDAVVYLVDAYDKERFAESKKELDALLSDEAL
ANVPFLILGNKIDIPYAASEEELRYHLGLTNFTTGKGKVNLADSNVRPMEVFMCSIVKKM
GYGEGFRWLSQYIK

>tr|A0A0K9R796|A0A0K9R796_SPIOL GTP-binding nuclear protein OS=Spinacia oleracea OX=3562 GN=SOVF_098770 PE=3 SV=1
MALPNQQTVDYPSFKLVLVGDGGTGKTTFVKRHLSGEFEKKYEPTIGVEVHPLDFHTNCG
QIRFYCWDTAGQEKFGGLRDGYYIHGQCAIIMFDVTARLTYKNVPTWHRDLCRVCENIPI
VLCGNKVDVKNRQVKAKQVTFHRKKNLQYYEISAKSNYNFEKPFLYLARKLAGDQNLH
FVESPALAPPEVQIDLAEQQRHEQELAVAAAQPLPDDDDDAFE

>tr|A0A803LTG6|A0A803LTG6_CHEQI Uncharacterized protein OS=Chenopodium quinoa OX=63459 PE=3 SV=1
MRQCLIYDSPEADARLIGIEYIVSEDLFMTLPDAEKRLWHSHEYEVKSGVLFLPGVPGAM
QRPDLAKVAQTYGKTIHFWQVDRGDNLPLGLPQIMMALTRDGQLYTHLASDVEKKYNV
NFEKERENRAYMKGLEHGIHPLANAEGKGLRTELREVDCHGGAGAHSTPRVFV

>tr|I0CC94|I0CC94_9CARY Thioredoxin-dependent peroxiredoxin OS=Tamarix hispida OX=189793 GN=Prx2 PE=2 SV=1

MACAAPTSAAVLSPSSSNPRAAGKLAAASIAKPFAQTLTAQNSFSGLRSSSSLRHFPLPASS
CRSSHSARRSFVVRAGELPLVGNEAPDFEAEAVFDQEFINVKLSDYRGKKYVILFFYPLDF
TFVCPTEITAFSDRCAEFEKLNTEVLGVSVDSVFSHLAWVQTDRKSGGLGDLKYPLISDVT
KSVSKAYNVLIPDQGIALRGLVIIDKEGIIQHSTINNLAIGRSVDETLRTLQALQYVQENPDE
VCPAGWKPGEKSMKPDPKLSKEYFAAI

>tr|A0A7C8Z5R6|A0A7C8Z5R6_OPUST Uncharacterized protein OS=Opuntia streptacantha OX=393608 PE=3 SV=1

MFLVDWFYGILASLGLWQKEAKILFLGLDNAGKTTLLHMLKDERLAQHQPTQYPTSEELS
IGRIKFKAFDLGGHQIARRVWKDYYAQVDAVVYLVDAFDRERFAESKRELDALLSDESLA
KVPFLILGNKIDIPYAASEEELRYALGLMNCTSGKGTVNLEGTNVRPLEVFMCSIVRKMGY
GEGFKWMSQYIK
